# Supplementary material for: Amyloid Beta Peptide (Aβ1-42) Reverses the Cholinergic Control of Monocytic IL-1β Release
Source: J Clin Med. 2020 Sep 7;9(9):2887. doi: 10.3390/jcm9092887 (PMC7564705; doi:10.3390/jcm9092887)
Supplement: Supplementary file 1 [file jcm-09-02887-s001.pdf]

Article

Amyloid Beta Peptide (A$\beta_{1\text{-}42}$) Reverses the Cholinergic Control of Monocytic IL-1β Release

Katrin Richter [1,*,†], Raymond Ogiemwonyi-Schaefer [1,†], Sigrid Wilker [1], Anna I. Chaveiro [1], Alisa Agné [1], Matthias Hecker [2], Martin Reichert [1], Anca-Laura Amati [1], Klaus-Dieter Schlüter [3], Ivan Manzini [4], Günther Schmalzing [5], J. Michael McIntosh [6,7,8], Winfried Padberg [1], Veronika Grau [1,†] and Andreas Hecker [1,*,†]

Supplemental Table S1: Lactate dehydrogenase (LDH) concentrations in cell culture supernatants of human monocytic U937 cells.

Reagents	Mean ± SD	n
/	**2.6** ± 1.2 %	15
LPS	2.9 ± 1.3 %	15
LPS, A$\beta_{1\text{-}42}$ 1 µM	1.0 ± 0.0 %	4
LPS, A$\beta_{1\text{-}42}$ 5 µM	0.7 ± 1.5 %	4
LPS, A$\beta_{1\text{-}42}$ 10 µM	1.7 ± 1.0 %	4
LPS, A$\beta_{42\text{-}1}$ 1 µM	4.0 ± 1.2 %	4
LPS, A$\beta_{42\text{-}1}$ 5 µM	4.5 ± 1.3 %	4
LPS, A$\beta_{42\text{-}1}$ 10 µM	5.5 ± 1.3 %	4
LPS, BzATP	2.4 ± 1.5 %	15
LPS, BzATP, ACh 7.5 µM	2.3 ± 1.6 %	6
LPS, BzATP, ACh 7.5 µM, A$\beta_{1\text{-}42}$ 0.5 µM	2.8 ± 1.2 %	6
LPS, BzATP, ACh 7.5 µM, A$\beta_{1\text{-}42}$ 1 µM	3.7 ± 0.8 %	6
LPS, BzATP, ACh 7.5 µM, A$\beta_{1\text{-}42}$ 2 µM	3.5 ± 1.0 %	6
LPS, BzATP, ACh 7.5 µM, A$\beta_{1\text{-}42}$ 5 µM	4.5 ± 1.3 %	4
LPS, BzATP, ACh 7.5 µM, A$\beta_{1\text{-}42}$ 10 µM	4.5 ± 0.6 %	4
LPS, BzATP, ACh 7.5 µM, A$\beta_{42\text{-}1}$ 1 µM	3.2 ± 1.5 %	4
LPS, BzATP, ACh 7.5 µM, A$\beta_{42\text{-}1}$ 5 µM	3.2 ± 0.5 %	4
LPS, BzATP, ACh 7.5 µM, A$\beta_{42\text{-}1}$ 10 µM	3.7 ± 2.2 %	4
LPS, BzATP, Nic 10 µM	3.2 ± 1.0 %	4
LPS, BzATP, Nic 10 µM, A$\beta_{1\text{-}42}$ 1 µM	4.0 ± 1.4 %	4
LPS, BzATP, Nic 10 µM, A$\beta_{1\text{-}42}$ 5 µM	5.7 ± 2.2 %	4
LPS, BzATP, Nic 10 µM, A$\beta_{1\text{-}42}$ 10 µM	6.2 ± 2.9 %	4
LPS, BzATP, ACh 10 µM	2.7 ± 1.0 %	4
LPS, BzATP, ACh 10 µM, A$\beta_{1\text{-}42}$ 5 µM	4.2 ± 1.3 %	4
LPS, BzATP, PC 100 µM	2.0 ± 1.4 %	4
LPS, BzATP, PC 100 µM, A$\beta_{1\text{-}42}$ 5 µM	3.2 ± 1.5 %	4
LPS, BzATP, CRP 5 µg/ml	1.7 ± 1.0 %	4
LPS, BzATP, CRP 5 µg/ml, A$\beta_{1\text{-}42}$ 5 µM	2.7 ± 0.5 %	4
LPS, BzATP, GPC 10 µM	3.8 ± 1.1 %	5
LPS, BzATP, GPC 10 µM, A$\beta_{1\text{-}42}$ 5 µM	8.0 ± 2.4 %	5
LPS, BzATP, DPPC 100 µM	1.4 ± 1.7 %	5
LPS, BzATP, DPPC 100 µM, A$\beta_{1\text{-}42}$ 5 µM	7.4 ± 1.7 %	5

U937 cells were primed with lipopolysaccharide (LPS) from *Escherichia coli* for 5 h. The P2X7 receptor agonist 2(3)-O-(4-benzoylbenzoyl)adenosine-5-triphosphate (BzATP; 100 µM) was added for another 30 min in the presence or absence of amyloid-β peptide (A$\beta_{1\text{-}42}$), reverse A$\beta_{42\text{-}1}$ and different nicotinic agonists. LDH release into the cell culture supernatant is given as % of the total release. ACh, acetylcholine; C-reactive protein, CRP; dipalmitoylphosphatidylcholine, DPPC; glycerophosphocholine, GPC; nicotine, Nic.

Journal of
Clinical Medicine

MDPI

Supplemental Table S2: Absolute values of interleukin-1β (IL-1β [pg/ml]) in cell culture supernatants of human peripheral blood mononuclear cells (PBMCs).

	IL-1β [pg/ml]												
**Reagents**	**LPS**	**LPS + BzATP**	**LPS + BzATP + PC**	**LPS + BzATP + PC + Aβ1-42**	**LPS + BzATP + PC + RgIA4**	**LPS + BzATP + PC +ArIB**	**LPS + BzATP + Aβ1-42**	**LPS + BzATP + RgIA4**	**LPS + BzATP + ArIB**	**LPS + PC**	**LPS + Aβ1-42**	**LPS + RgIA4**	**LPS + ArIB**
Donor 1	**8**	468	135	259	406	423	370	-	-	-	-	-	-
Donor 2	190	381	274	486	311	-	471	419	458	-	-	-	-
Donor 3	49	240	7	149	148	247	384	206	429	26	41	31	40
Donor 4	48	234	26	505	361	299	427	378	681	42	101	67	71
Donor 5	149	1307	203	1312	1299	1298	1067	1284	1263	90	82	168	168
Donor 6	110	2167	99	775	1044	1577	674	1518	2079	52	65	54	72
Donor 7	333	2332	631	1980	2221	2092	1544	1955	2421	156	182	179	166
n =	7	7	7	7	7	6	7	6	6	5	5	5	5
Median	110	468	135	505	406	861	471	852	972	52	82	67	72

Human PBMCs were primed with lipopolysaccharide (LPS) from *Escherichia coli* (5 ng/ml; LPS-pulse). The P2X7 receptor agonist 2(3)-O-(4-benzoylbenzoyl)adenosine-5-triphosphate (BzATP; 100 µM) was added for another 30 min in the presence or absence of amyloid-β peptide (Aβ1-42; 5 µM), phosphocholine (PC; 200 µM) or conopeptides [V11L, V16D]ArIB (ArIB; 500 nM) and RgIA4 (50 nM). IL-1β release into the cell culture supernatant is given as pg/ml.

Journal of
Clinical Medicine

Supplemental Table S3: Lactate dehydrogenase (LDH) concentrations in cell culture supernatants of human peripheral blood mononuclear cells (PBMCs).

Reagents	Mean ± SD	n
LPS	2.4 ± 2.8 %	7
LPS, PC 200 µM	1.8 ± 1.5 %	5
LPS, $A\beta_{1-42}$ 5 µM	2.0 ± 1.9 %	5
LPS, RgIA4 50 nM	1.2 ± 0.8 %	5
LPS, ArIB 500 nM	1.2 ± 1.1 %	5
LPS, BzATP	5.1 ± 2.0 %	7
LPS, BzATP, DMSO 0.5 %	5.5 ± 2.5 %	4
LPS, BzATP, PC 200 µM	2.8 ± 1.8 %	7
LPS, BzATP, PC 200 µM, $A\beta_{1-42}$ 5 µM	6.3 ± 2.2 %	7
LPS, BzATP, PC 200 µM, RgIA4 50 nM	4.7 ± 1.6 %	7
LPS, BzATP, PC 200 µM, ArIB 500 nM	4.7 ± 1.0 %	6
LPS, BzATP, $A\beta_{1-42}$ 5 µM	5.8 ± 2.4 %	6
LPS, BzATP, RgIA4 50 nM	4.8 ± 1.7 %	6
LPS, BzATP, ArIB 500 nM	3.8 ± 2.5 %	6

Human PBMCs were primed with lipopolysaccharide (LPS) from *Escherichia coli* (5 ng/ml; LPS-pulse). The P2X7 receptor agonist 2(3)-O-(4-benzoylbenzoyl)adenosine-5-triphosphate (BzATP; 100 µM) was added for another 30 min in the presence or absence of amyloid-β peptide ($A\beta_{1-42}$; 5 µM), phosphocholine (PC; 200 µM) or conopeptides [V11L, V16D]ArIB (ArIB; 500 nM) and RgIA4 (50 nM). LDH release into the cell culture supernatant is given as % of the total release.

© 2020 by the authors. Submitted for possible open access publication under the terms and conditions of the Creative Commons Attribution (CC BY) license (http://creativecommons.org/licenses/by/4.0/).
